# Oxidative stress induced by Se-deficient high-energy diet implicates neutrophil dysfunction via Nrf2 pathway suppression in swine

**DOI:** 10.18632/oncotarget.14550

**Published:** 2017-01-07

**Authors:** Tianshu Yang, Zeping Zhao, Tianqi Liu, Ziwei Zhang, Pengzu Wang, Shiwen Xu, Xin Gen Lei, Anshan Shan

**Affiliations:** ^1^ Northeast Agricultural University, Harbin, P. R. China; ^2^ Department of Animal Science, Cornell University, Ithaca, NY, USA; ^3^ Key Laboratory of Animal Cellular and Genetic Engineering of Heilongjiang Province, Northeast Agricultural University, Harbin, China

**Keywords:** high-energy, se deficiency, neutrophils, oxidative stress, Nrf2

## Abstract

The mechanism of the interaction between Se deficiency and high energy remains limited. The aim of the current study was to identify whether Se-deficient, high-energy diet can induce oxidative stress, and downregulate the Nrf2 pathway and phagocytic dysfunction of neutrophils. We detected the phagocytic activity, ROS production, protein levels of Nrf2 and Nrf2 downstream target genes, and the mRNA levels of 25 selenoproteins, heat shock proteins, and cytokines in neutrophils. Cytokine ELISA kits were used to measure the serum cytokines. The concentration of ROS was elevated (*P* < 0.05) in obese swine fed on a low Se diet (less than 0.03 mg/kg Se) compared to control swine. The protein levels of Nrf2 and its downstream target genes were depressed during Se deficiency and high-energy intake. The mRNA levels of 16 selenoproteins were significantly decreased (*P* < 0.05) in the Se-deficient group and Se-deficient, high-energy group compared to the control group. However, the mRNA levels of 13 selenoproteins in peripheral blood neutrophils were upregulated in high energy group, except TrxR1, SelI and SepW. In summary, these data indicated that a Se-deficient, high-energy diet inhibits the Nrf2 pathway and its regulation of oxidative stress, and prompted a pleiotropic mechanism that suppresses phagocytosis.

## INTRODUCTION

The nutrient elements of animal diets are key to redox homeostasis and the functions of the body's immune system. When there is redox equilibrium dysfunction, the functions of the immune system are impaired as a result of the selenium and lipid-stimulated oxidants and antioxidants [1–3]. Several studies reported that Se deficiency disrupted the expression of selenoproteins in many tissues and organs of livestock and humans [4, 5]. In these situations, immune system dysfunction is caused by oxidative stress and the generation of ROS. Selenoproteins, such as GPX, TRXR, SEPP1, SelN and SelW, exert antioxidative ability [6]. High-energy diet results in obesity, the continuous generation of ROS from the stimulation of a metabolic surplus, and a drop in the immune function of the body by remaining in a persistent condition of oxidative stress. This induces low-grade, chronic inflammation from the generation of lipid peroxidation and enhances the expression quantity of pro-inflammatory cytokines, such as IL-1, IL-6, IL-10, TNF-α, and COX-2 [7, 8].

The nature of oxidative stress underscoring the production of ROS can be induced by external factors. In homeostatic conditions, Nrf2, which has no functional activity, exists in the cytoplasm and combines with Keap1. In conditions of oxidative stress, Nrf2 dissociates from Keap1, moves to the nucleus, and combines with ARE to form Nrf2-ARE, activating downstream extranuclear genes such as *sod2, gpx1, trxR1*, *ho-1* and catalase [9]. The Nrf2 pathway has been shown to have both up- and down-regulated functions [10]. Nrf2 pathway could also suppress potent phagocytic activity [11, 12]. In studies in the bone marrow, the expression of glutathione in immature dendritic cells decreased with damaged phagocytosis and without Nrf2 in the bone marrow [13]. The Nrf2 pathway can be adjusted by many factors, such as the intake of Se and energy. Nrf2 will stimulate the expression of up and downstream genes through relevant metabolic pathways [14]. At present, as shown in human and mouse experiments, Se deficiency activated the Nrf2 pathway. This increased the expression of Nrf2 so that it could increase the expression of relevant downstream antioxidant genes, including selenoproteins GPX1 and TRXR-1 [15]. In relevant experiments looking at high-fat intake, fatty liver induced by high-fat diet suppressed the Nrf2 pathway, whose main inhibition mechanism might be associated with a chronic pathological process [16]. The previous research also indicated that the inhibition factors about Nrf2 pathway also included high blood pressure, high-glucose, premature aging and zinc deficiency [17–19]. When the Nrf2 pathway was restrained, the body's antioxidant ability decreased, leading to the promotion of oxidative stress and the dysfunction of the immune system.

Invading pathogens can be killed by neutrophils through phagocytosis and the release of inflammatory factors (IL-1β, IL-6, IL-8, COX-2) [20]. Neutrophil activation generates large quantities of ROS to activate the Nrf2 pathway and, as a result, the function of neutrophils is feedback inhibited [21, 22]. Related research in mice fed with high-fat diets and with a knockout in ApoE found that inflammatory monocytes and neutrophils could be regulated by nestin (+) BM cells, indicating that these cells could regulate inflammatory cells during chronic inflammation [23]. Similar results were observed in wild-type mice fed high-fat diets. Monocyte-depletion analyses were performed, showing that neutrophils were preferentially recruited to the femoral artery, and proved that PMN-derived MCP-1 was important in the accumulation of leukocytes [24]. The dysfunction of neutrophils is associated with a Se-deficient diet. However, the effect of Se-deficiency and a high-energy diet on neutrophil dysfunction is still not clear. We hypothesized that Se-deficiency and high-energy diets result in a phagocytosis problem in neutrophils by restraining the Nrf2 pathway from producing persistent oxidative stress, resulting in the further dysfunction of the immune system. Herein, the influence and mechanism on neutrophils associated with a Se-deficient, high-energy diet can be clarified by monitoring phagocytosis activity, and the concentration of reactive oxygen. To further link ROS generation to neutrophil phagocytosis, we evaluated the level of Nrf2, the downstream genes, 25 selenoproteins, HSPs and relevant cytokines, which were acquired from swine fed with a Se-deficient, high-energy diet.

## RESULTS

### Se-deficient high-energy diet inhibited neutrophil phagocytosis

To confirm whether Se deficiency and high-energy diets affect the function of neutrophils, swine neutrophils were freshly isolated from a control group, Se^−^ group, energy^+^ group, and Se^−^-energy^+^ group. As expected, the Se-deficient and high-energy treatment prohibited the ability of neutrophils to endure phagocytosis compared with the control group (Table 1). Compared to the control group, dietary Se deficiency significantly decreased the neutrophils phagocytic activity (*P* < 0.05) as well as the OD phagocytic activity index, which was decreased (*P* < 0.05). Supplementation of energy resulted in a decreased percentage of phagocytosis compared with the control group, and no significant differences between the energy^+^ group and Se^−^ group were observed in these parameters. These data demonstrate a critical role for Se^−^ and energy^+^ in regulating neutrophil phagocytosis.

**Table 1:** Mean values within a row with different letters are significantly different (P < 0.05)

	CD	SDD	HED	SED
Phagocytic activity (%)^1^	**64.87 ± 0.287^C^**	**49.54 ± 0.339^Aa^**	**55.46 ± 0.462^Bb^**	**43.77 ± 0.138^Ab^**
Index of phagocytic activity^2^	**1.47 ± 0.119^C^**	**1.24 ± 0.213^Aa^**	**1.26 ± 0.193^Aa^**	**1.16 ± 0.088^Bb^**

^1^Number of white cells containing at least three engulfed particles per 100 white cells (neutrophils).

^2^Number of engulfed particles per total number of neutrophils observed.

### Se-deficient high-energy diet promoted the generation of ROS in neutrophils

**Figure 1:**
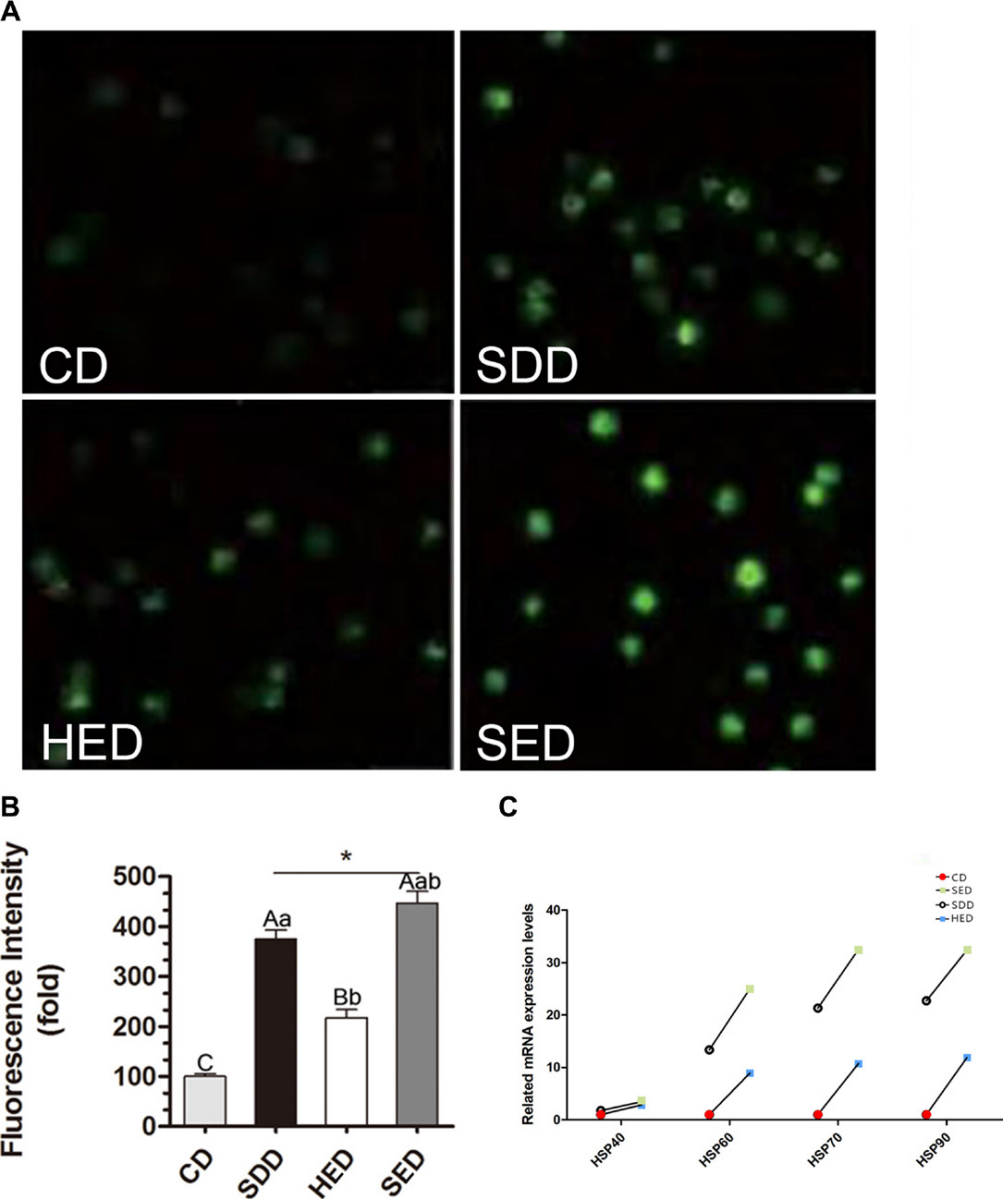
Se-deficient diet, high-fat diet and Se-deficient, high-energy diet induced ROS levels in neutrophils (**A**) Immunofluorescence analysis was performed using DCFH-DA (green fluorescence, 5 mM). Neutrophils were visualized using fluorescence microscopy. (**B**) Three different fields were randomly counted for green positive neutrophils using Image J and the average fluorescence intensity of cells in eac diet were compared to that of control. Statistical significance was calculated by one-way ANOVA test, where bars that are significantly different (*p* < 0.05) from each other do not share the same letter. The data were expressed as the mean ± SD (*n* = 10). (**C**) Neutrophils mRNA levels of Hsp40, Hsp60, Hsp70 and Hsp90 in different treated groups. Bars that do not share the same letters are significantly different (*p* < 0.05) from each other. The data were expressed as the mean ± SD (*n* = 10).

To assess the reason why neutrophil phagocytosis is decreased by Se and energy status, we evaluated the degree of oxidative stress by detecting the production of ROS in swine neutrophils. Figure 1A shows typical images of ROS under various conditions. As shown in Figure 1B, a Se-deficient, high-energy diet significantly increased ROS production 447 ± 23.56-fold compared with the control group. Treatment with a Se-deficient and high-energy diet showed a similar, strengthened effect on ROS production, induced from 100 ± 5.64-fold over the control to 375 ± 17.81-fold and from 100 ± 5.64-fold over the control to 217 ± 16.73-fold, respectively.

Furthermore, we determined whether there was a change in the heat shock protein (HSP) gene expression in neutrophils. The mRNA levels of Hsp40, Hsp60, Hsp70 and Hsp90 were significantly increased in the low Se and high-energy groups (*P* < 0.05) compared with the corresponding control group (Figure 1C). Specifically, the mRNA levels of Hsp60, Hsp70 and Hsp90 increased ~2000% due to the high-energy and low Se diet feeding. HSPs showed the highest expression level among all the groups. Notably, the lowest range of Hsp40 mRNA expression during the Se-deficient and high-energy condition was observed.

### Se-deficient high-energy diet influenced the mRNA expression of selenoproteins

**Figure 2:**
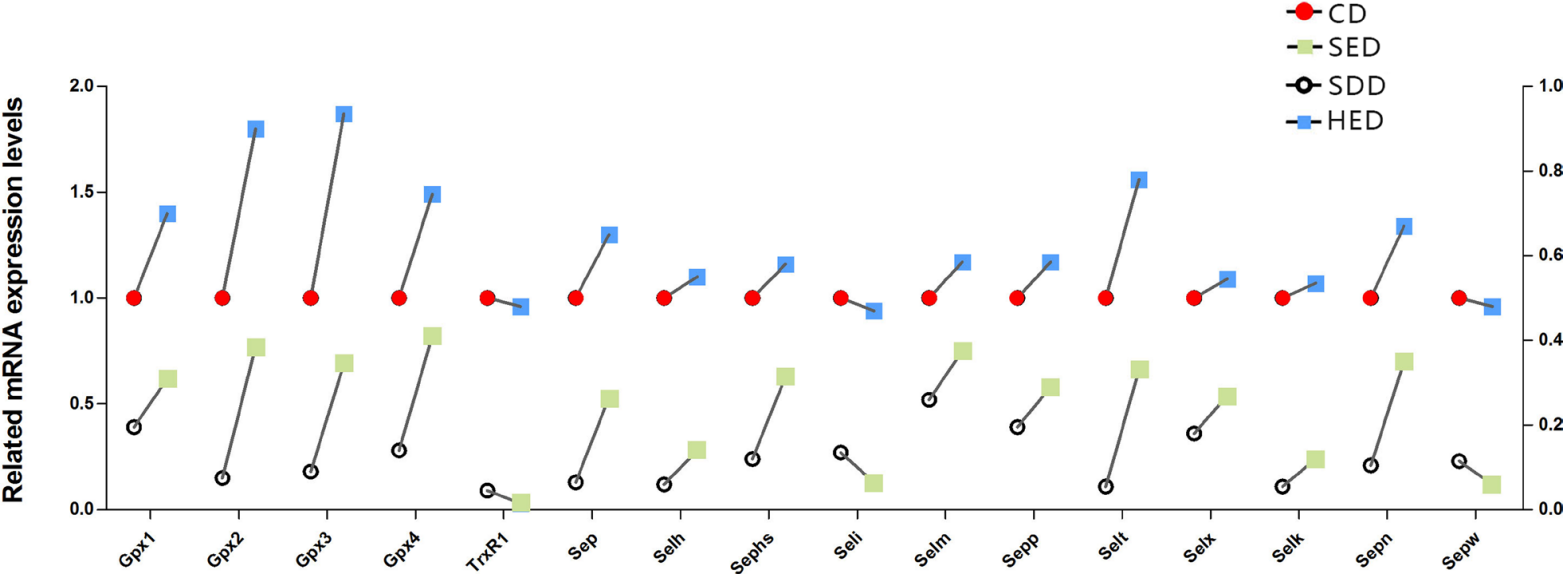
Neutrophils mRNA levels of selenoproteins genes (TrxR1, Gpx1, Gpx2, Gpx3, Gpx4, Sep, Selh, Sephs, Seli,Selm, Sepp1, Selt, Selx, Selk, Sepn and Sepw) in the different treated regions Bars that do not share the same letters are significantly different (*p* < 0.05) from each other. The data were expressed as the mean ± SD (*n* = 10).

We detected the mRNA expression of 25 selenoproteins. However, the remaining 9 selenoproteins were expressed faintly in neutrophils. As shown in Figure 2, the mRNA abundances of the selenoproteins (TRXR-1, GPX1, GPX2, GPX3, GPX4, SeP, SelH, SEPHS, SelI, SelM, SEPP1, SelT, SelX, SelK, SepN, SepW) were significantly decreased (*P* < 0.05) in the Se^−^ group and Se^−^-energy^+^ group compared to the control group in swine peripheral blood neutrophils. However, surprisingly, the levels of 13 selenoproteins were regulated in an opposite fashion in the energy^+^ group, except TRXR-1, SelI and SepW. Dramatically, the results showed that the deficiency of dietary Se and high-energy intake have the opposite effect on regulating selenoprotein profiles in neutrophils. Interestingly, the GPX family levels were more sensitive to the Se and energy status amongst the Se^−^ group, the energy^+^ group and Se^−^-energy^+^ group. Obese pigs with the low Se diet had neutrophil concentrations of GPX1, GPX2, GPX3 and GPX4 that were 38%, 28%, 31% and 18% lower (*P* < 0.05) than the control group, respectively. Thus, the range of the mRNA expression of selenoproteins in the control group and Se- group are similar to energy^+^ group and Se^−^-energy^+^ group.

### Se-deficient high-energy diet downregulated Nrf2 and its downstream target genes

**Figure 3:**
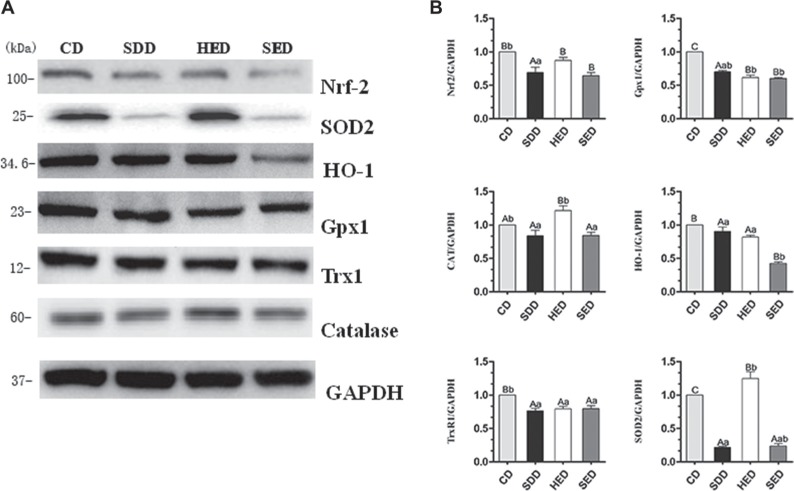
CD, SDD, HED and SED treatment for decreases in Nrf2-regulated antioxidant proteins in neutrophils (**A**) Western blot analyses of Nrf2 regulated antioxidant proteins (Nrf2, TrxR1, HO-1, GPX1, catalase and SOD2) in SDD, HED and SED treated neutrophils. (**B**) Relative intensity of the protein signal was calculated using Image-J software and normalized to GAPDH. Statistical significance was calculated by a one way ANOVA test, where bars are significantly different (*p* < 0.05) from each other do not share the same letter. The data were expressed as the mean ± SD (*n* = 10).

To determine whether the Nrf2 pathway regulated oxidative stress under a situation of Se deficiency and high-energy, we evaluated the protein level of Nrf2 and the main downstream target genes (SOD2, HO-1, Gpx1, TrxR1, catalase) in the Nrf2 pathway. Protein levels of Nrf2 were decreased in the Se deficiency, high-energy and Se-deficient, high-energy treatment compared to the control group (Figure 3). As expected, the differential expression of five down-regulated genes (SOD2, HO-1, Gpx1, TrxR1, and catalase) was observed in the Se-group and Se^−^-energy^+^ group. These data indicate that Se^−^ and Se^−^-energy^+^ treatment downregulated Nrf2 and Nrf2 downstream target genes. However, protein expression of the three down-regulated genes (SOD2, Gpx1, and TrxR1) and two up-regulated genes (HO-1, catalase) was examined in the energy^+^ group. Taken together, these data show that Se and energy status reduced protein level of Nrf2 and inhibit downstream proteins of the Nrf2 pathway compared with the control group.

### Se-deficient high-energy diet changed the expression of cytokines in serum

**Figure 4:**
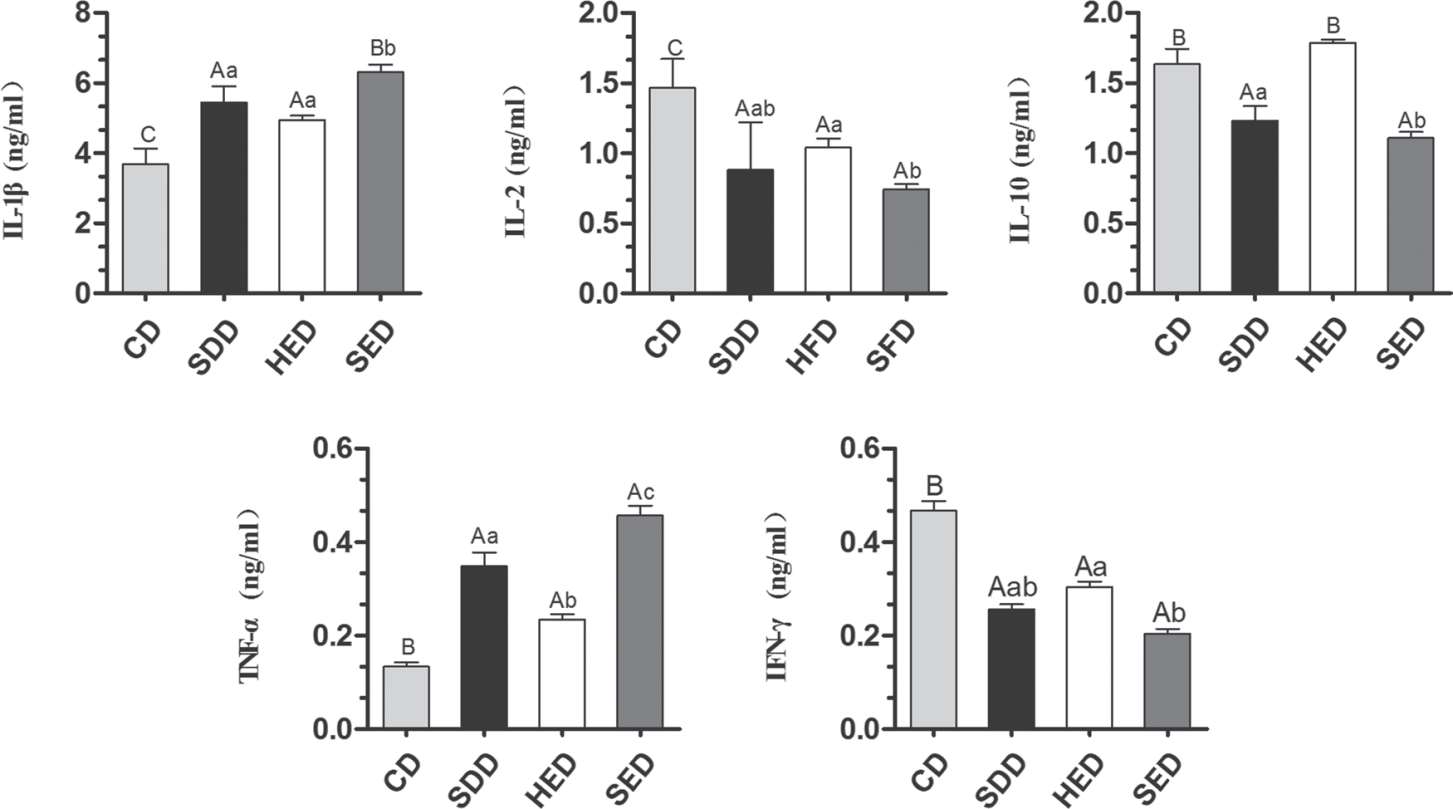
Different diets induce cytokines activation in serum (**A**) Cytokines (IL-1β, IL-2, IL-10, TNF-α and IFN-γ) in serum extracts was measured by ELISA. (**B**) Relative intensity of the cytokines levels was calculated using GraphPad Prism. Bars that do not share the same letters are significantly different (*p* < 0.05) from each other. The data were expressed as the mean ± SD (*n* = 10).

To evaluate whether a Se-deficient, high-energy diet induced inflammation response in the body, we examined the level of cytokines in serum by ELISA. As shown in Figure 4, the level of IL-1β and TNF-α in the serum elevated ~3.0 and ~0.3-fold, respectively, with Se^−^, energy^+^ and Se^−^-energy^+^ treatment, which are consistent with the mRNA data in the neutrophils. However, the level of IFN-γ and IL-2 significantly decreased (*P* < 0.05) in the Se^−^, energy^+^, and Se^−^-energy^+^ groups compared to the control group. The expression of IL-10 was reduced by Se deficiency and Se-deficient, high-energy treatment, but the energy^+^ group significantly increased its level from 1.638 ± 0.109 to 1.786 ± 0.025-fold.

### Se-deficient high-energy diet upregulated mRNA expression of inflammatory factors

**Figure 5:**
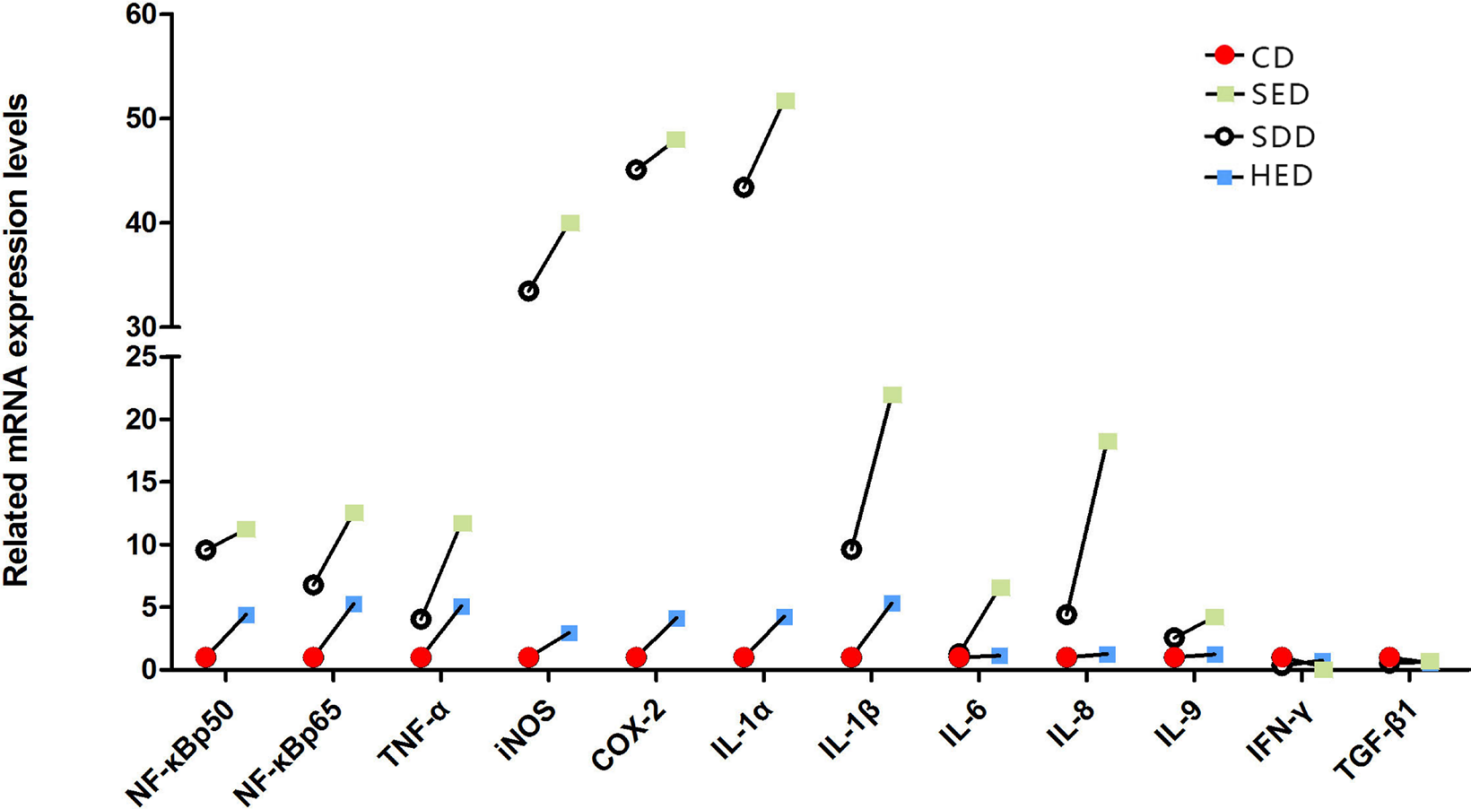
Neutrophil mRNA levels of inflammatory factors genes (IL-1α, IL-1β, IL-6, IL-8, IL-9, Nf-κbp50, Nf-κbp65, TNF-α, iNOS, COX-2, IFN-γ and TGF-β1) in different treated groups Bars that do not share the same letters are significantly different (*p* < 0.05) from each other. The data were expressed as the mean ± SD (*n* = 10).

The mRNA levels of IL-1α, IL-1β, IL-6, IL-8, IL-9, Nf-κbp50, Nf-κbp65, TNF-α, INOS, and COX-2 were elevated in the neutrophils of the Se^−^, energy^+^, and Se^−^-energy^+^ groups compared with the corresponding control group (Figure 5). In the Se^−^-energy^+^ group, the mRNA expression level of IL-1β, IL-6 and IL-8 were 128%, 405% and 313% (*P* < 0.05) greater than Se^−^ group, respectively. The rest of the genes showed an opposite, downward fashion, with the obvious decline in IFN-γ and TGF-β1. Inflammation-related gene expression was highly stimulated in the high-energy and low Se feeding group. Of these, IL-1α, iNOS and COX-2 showed the highest responsiveness in neutrophils of the Se deficiency and high-energy condition.

## DISCUSSION

Nutrient elements act in balance with oxidation metabolism and immune system function. For instance, Se deficiency leads to reduced selenoprotein content in tissues and organs [4, 5, 25] and results in chronic inflammation, which is induced by oxidative stress [26], which in turn hampers immunity. These changes will also impair the functions of immune cells [27]. In addition, a high energy content diet would give rise to obesity and induce chronic oxidative stress, which would further bring about chronic inflammation [28] and disrupt the functioning of immune cells [29]. This study proved that a Se-deficient, high-energy diet can inhibit the Nrf2 pathway in neutrophils, trigger sustaining oxidative stress and reduce phagocytosis. Moreover, a Se-deficient, high-energy daily ration can cause chronic inflammation in the body.

Many references indicated that the oxidative stress of tissues and organs induced by the Se-deficient diet might be associated with selenoproteins [30–33]. The rationale is that the reduced expression of antioxidative selenoproteins may lead to redox homeostasis disorder and ROS accumulation, which encourages oxidative stress [6]. Studies on neutrophils in Se-deficient broilers found that a shortage of selenium in daily rations lifted the level of Hsp40, Hsp60, Hsp70, Hsp90, iNOS and NO production by a large margin [34]. In our experiment, Se deficiency diet lowered the expression of 16 selenoproteins, but boosted Hsp40, Hsp60, Hsp70 and Hsp90. The level of ROS also increased, suggesting that Se-deficient diet was responsible for oxidative stress in the peripheral neutrophils of swine. Furthermore, the high-energy diet involved in this experiment improved the expression of 13 selenoproteins in neutrophils, TRXR-1, SelI, and SepW excluded, which was partly consistent with the findings of our previous research. The latter tested the level of selenoproteins in pigs on high-fat diets and discovered that excessive fats enhanced 12 selenoproteins, respectively, in six types of tissues. Additionally, the expression of selenoproteins in different types of tissues was reduced. Additionally, the expression of TRXR-1 in the pancreas, hypothalamus and pituitary dropped [35], indicating that exorbitant fats are tissue-specific for the expressions of selenoproteins. High-energy daily rations could cause obesity. Obesity likely generates increased blood lipids and ROS. The supposition that oxidative stress is attributed to high-energy diets has been confirmed by experiments on humans and a variety of animals [36–39]. *In vitro* experiments have demonstrated that fatty acids will bring about cellular oxidative stress [40–42]. *In vitro* cultured bovine hepatocytes applied with non-esterified fatty acids (NEFAs) hindered the expression of Nrf2 through the activation of ROS-p38-p53/Nrf2 signaling pathway, and, as a result, induced intracellular oxidative stress [43]. What's more, high-energy diets could impede the production of antioxidants and jeopardize the redox homeostasis by directing the Nrf2 signaling pathway to induce oxidative stress [44]. This experiment proved that a low level of Se and high content of energy prompts the production of ROS in neutrophils and triggers oxidative stress.

Some studies have verified that Nrf2 represents the center regulator of oxidative stress, which is normally located in the cytoplasm and dissociates with Keap1. In the case of cellular oxidative stress, once the system is activated, Nrf2 would enter the nucleus and bind with ARE to activate the downstream antioxidant genes of the Nrf2 pathway to restore the dynamic redox homeostasis [39]. The present experiment has shown that detected Nrf2 proteins were on the decline as the level of SOD2, GPX1, TRXR-1, HO-1, other downstream genes of the Nrf2 pathway and catalase proteins decreased. Because the expression of antioxidant genes was inhibited and substantial ROS could not be reduced, then sustained and unregulated oxidative stress was observed in neutrophils. Relevant studies demonstrated that many factors account for the inhibition of the Nrf2 pathway, such as chronic inflammatory diseases and hypertension controlled by obesity [16, 17]. Moreover, studies on Hutchinson-Gilford progeria syndrome (HGPS) found that the Nrf2 pathway participated in the oxidative stress induced by a high-fat diet [44]. That coincides with our results, which show that high-energy diet causes oxidative stress in neutrophils and obstructs the Nrf2 pathway. Studies on humans and mice taking in inadequate Se found that a low amount of Se activated the Nrf2 pathway and promoted the expression of the selenoprotein, TRXR-1, while reducing the amount of some selenoproteins in multiple organs [14, 45]. That was contrary to what was observed in this experiment. The conclusion that the expression of TRXR-1 improved disagreed with most relevant reports that Se deficiency would decrease the content of selenoproteins [46–48]. It is noteworthy that the expression of TRXR-1 reduced significantly in the Se^−^, energy^+^, and Se^−^-energy^+^ group and that the reduction peaked in the first group, implying that TRXR-1 probably participates in the Se-deficient inhibition of the Nrf2-pathway. On the one hand, the inhibited Nrf2 pathway induced oxidative stress in neutrophils. On the other hand, the decreased expression of selenoproteins provoked oxidative stress in neutrophils.

As the neutrophils act as an important defender of the peripheral immune system, its intracellular oxidative stress is closely related to phagocytosis. When phagocytosis occurs, oxidative burst occurs inside cells, along with the emergence of a vast number of ROS [49], propelling the Nrf2 pathway to produce antioxidants to down regulate oxidative burst and maintain the dynamic redox homeostasis [50]. While observing obese individuals, we found that macrophages infiltrated the adipose tissue and their phagocytosis deteriorated [51]. A low-selenium diet would exert similar impacts on neutrophils, as was demonstrated by Hogan JS, who studied the relationship between the Se content in cow's fodder and function of neutrophils in the 1990s, concluding that deficiency of Se could impair the phagocytic ability of neutrophils [52]. In this study, the Se-deficient, high-energy diet inhibited the activation of the Nrf2 pathway in neutrophils and the reduction of accumulated ROS, keeping neutrophils in a constant state of oxidative stress. Therefore, dysfunction of neutrophil phagocytosis might be impaired by the threshold of free oxygen radicals.

Neutrophils activated by oxidative stress generated a substantial amount of pro-inflammatory cytokines, including IL-6, IL-8, COX-2, and TNF-α, and undermined the expression of anti-inflammatory factors, such as IFN-γ and TGF-β1 [20]. This was in line with what happened to neutrophils in our study. Studies on small-molecule organo-selenium found that its levels were related with chronic low-grade inflammation [53]. Studies on poultry and mice fed low Se diets also revealed that a low content of Se could induce inflammation in some organs and tissue [54, 55]. Apart from that, obesity caused by high-fat would occasionally cause chronic low-grade inflammation characterized by macrophage infiltration in the adipose tissue where changes in relevant inflammatory factors, such as TNF-α and IL-1, were also detected [56]. Different behaviors of serum cytokines in the Se^−^, energy^+^, and Se^−^-energy^+^ group did indicate that both a low level of Se and a high content of energy might result in chronic inflammation that would subject neutrophils to continuous exposure to oxidant and pro-inflammatory factors [57, 58]. In this way, oxidative stress in neutrophils would continue and the accumulated ROS would do harm to cell structures [59], bringing extra damage to neutrophil phagocytosis.

**Figure 6:**
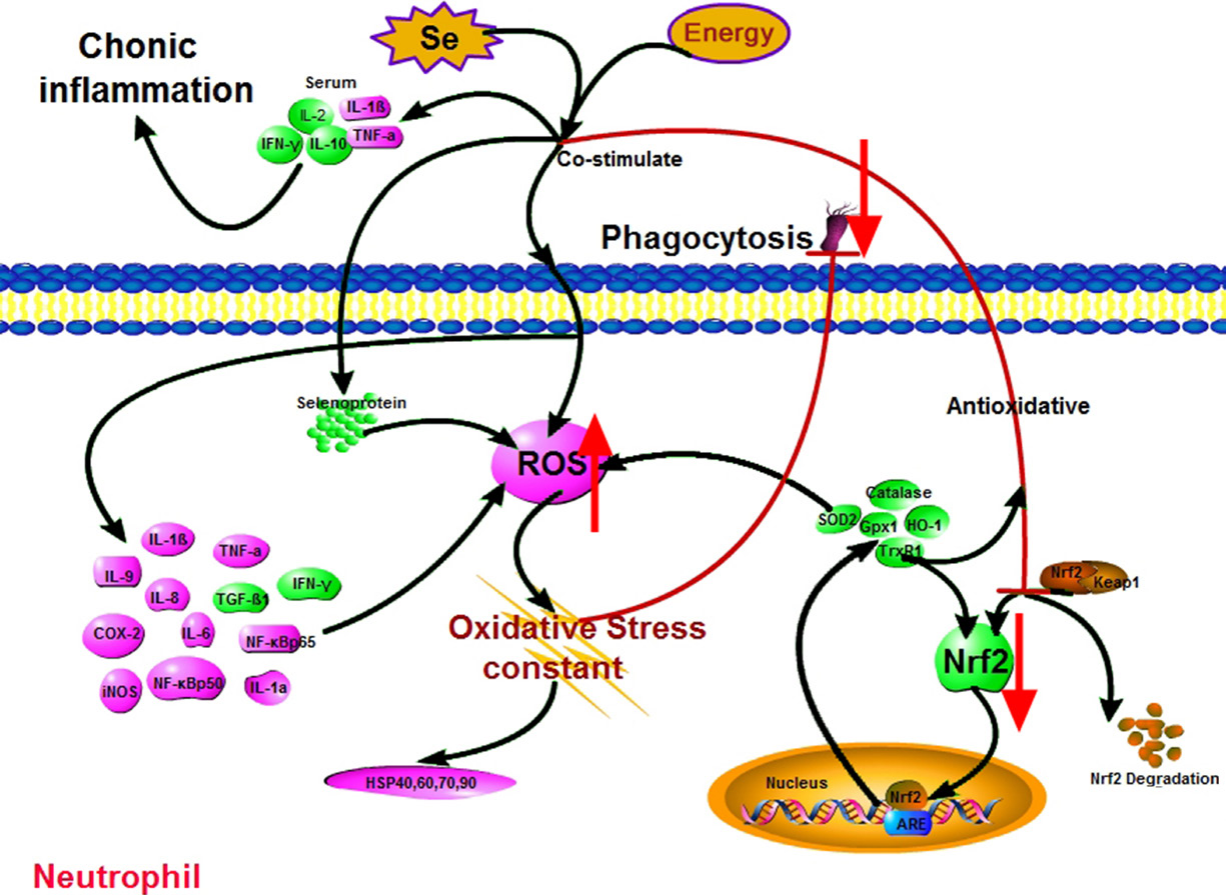
Scheme shows mechanism of Nrf2 pathway-oxidative stress modulation due to Se^-^/energy^+^ treatment Pink denotes up-regulation (neutrophils: ROS, HSPs, IL-1α, IL-1β, IL-6, IL-8, IL-9, TNF-α, COX-2, iNOS, Nf-κbp50 and Nf-κbp65; serum: IL-1β and TNF-α); green denotes down-regulation (neutrophils: Nrf2, SOD2, HO-1, catalase, Selenoproteins, IFN-γ and TGF-β1; serum: IL-2, IL-10 and IFN-γ).

In conclusion, through the histological investigation of neutrophil function against oxidative stress, this study has shown that the activation of the Nrf2 pathway in neutrophils can be inhibited by Se-deficient, high-energy diet. This further damages phagocytosis via chronic oxidative stress, as well as causing chronic inflammation in the body (Figure 6). Specifically, how Se deficiency and high-energy diet are involved in the Nrf2 pathway is not clear. We demonstrated that persistent oxidative stress is the key to impaired phagocytosis. The mechanistic basis of this difference remains to be explored.

## MATERIALS AND METHODS

### Treatment of experimental animals

All procedures used in our study were approved by the Institutional Animal Care and Use Committee of Northeast Agricultural University. A 2 × 2 design trial was conducted that involved two dietary energy levels (normal DE v. high DE) and two dietary Se supplementation levels (0 and 0.3 mg/kg Se in the diet, the basal diet contains less than 0.03 mg/kg Se by analysis) (Supplementary Table 1). A total of 40 crossbred (Duroc x Landrace x Yorkshire, uncastrated) swine, with an average BW of 10 ± 0.72 kg, were randomly allotted to five groups (*n* = 10). All groups were fed with a Corn-Soybean meal diet, which was divided into three stages according to the growth stage of swine, stage I diet (10~30 kg BW), stage II diet (30~60 kg BW) and stage III diet (60~110 kg BW). Some swine were maintained either on a Se-deficient diet (Se^−^ group, SDD) with the basal diet, or on a sodium selenite diet (control group, CD). The other swine were maintained either on a high-energy diet (energy^+^ group, HED) containing fat energy/digestion from 16%~30%, or on a Se-deficient, high-energy diet (Se^−^-energy^+^ group, SED) supplemented with 0 mg/kg Se and fat energy/digestion 16%~30%. The basal diet contains 3296 kJ/kg DE (stage I diet), 3294 kJ/kg DE (stage II diet), and 3303 kJ/kg DE (stage III diet). The high-energy diet contains 3492 kJ/kg DE (stage I diet), 3688 kJ/kg DE (stage II diet), and 3929 kJ/kg DE (stage III diet). Swine were euthanized after 16 weeks in the experiment, and the peripheral blood neutrophils were extracted for inspection. The neutrophils were frozen in liquid nitrogen with Trizol immediately and stored at −80°C until required.

### Preparation of swine peripheral blood neutrophils suspension

Fifteen milliliters of fresh porcine vein blood was mixed with sodium citrate tribasic in a ratio of seven to one and mixed into 15 ml of PBS. The mix was added to the 15 ml of neutrophil separation medium and centrifuged at 500xg for 20 min. The second and third layer were collected into a tube containing 15 ml of PBS, mixed well, and centrifuged at 250xg for 10 min. After centrifuging, the supernatant was discarded and cells at the bottom of the tube were collected. The cells were mixed with 10 ml of PBS. The mixed liquid was added into the lymphocyte separation medium and centrifuged at 500xg for 20 min. Cells were recovered from the tube and were washed and re-suspended in PBS. Swine peripheral blood neutrophils were collected with 1 ml of Trizol and stored immediately at −80°C for the RNA isolation.

### Assessment of phagocytosis by neutrophils

Phagocytic activity of swine neutrophils was measured with hydrophilic MSHP by a direct microscopic counting procedure, using a modified method described by Vetvicka et al., [60]. Blood smears were prepared and stained in accordance with methods explained by May-Grünwald and Giemsa-Romanowski [61]. One hundred neutrophils were examined to observe the phagocytic activity of neutrophils containing at least 3 engulfed particles in each smear. The phagocytic activity index was calculated as engulfed particles per total number of phagocytes observed. Increased intracellular fluorescence corresponds to increased phagocytosis.

### Measurement of ROS

The concentration of separated neutrophils was adjusted to 106/ml by centrifuging 1 ml of cell suspension at 500xg for 5 min. After centrifuging, the supernatant was discarded, cells at the bottom of the tube were collected, and then the neutrophils were washed with PBS twice. After washing, 1 μl of DCFH-DA fluorescent probe was added in the mix of neutrophils, incubated at 37°C for 30 min, and washed with PBS. Then, 0.1 ml of cell suspension was smeared, captured under a fluorescence microscope, and ROS-associated fluorescent signals were quantified with Image J software (Broken Symmetry Software, Albert, Cardona) [62]. The relative intensities were reported in arbitrary units per cell.

### Western blot analysis

Total protein was assessed with SDS-polyacrylamide gel electrophoresis under reducing conditions on 12% gels and then transferred to nitrocellulose membranes using a tank transfer at 80 mV in Tris-glycine buffer containing 20 % methanol for 2 h. Nitrocellulose membranes were blocked for 2 h with 5 % skim milk at 37°C and incubated for 12 h with diluted primary swine antibody Nrf2 (1:1000), SOD2 (1:1000), HO-1 (1:1000), Gpx1 (1:1000), TrxR1 (1:1000) and Catalase (1:1000) at 4°C. This was then followed incubated with a horseradish peroxidase-conjugated secondary antibody against rabbit IgG (1:1500, Santa Cruz, CA, USA) using the ECL kit (Kangweishiji Biotechnology, Beijing, China). For statistical analysis, a box plot analysis was applied.

### ELISA based measurement of cytokines activity

The concentration of serum cytokines (IL-1β, IL-2, IL-10, TNF-α, IFN-γ) was determined using an ELISA kit (Hermes Criterion Biotechnology, Canada). The level of substance was detected in the sample kit using a double antibody sandwich method. Microtiter plate wells were coated with monoclonal antibody. The test substance was then added, and combined with the HRP-labeled test substance antibody, becoming antibody-antigen-enzyme labeled antibody complexes. After washing completely, TMB substrate was added. HRP-catalyzed reactions are blue, and turn yellow as the reaction progresses. The color in a sample substance was positively correlated with the amount of substance. We determined the concentration of a substance in the sample by comparing the absorbance at 450 nm wavelength, calculated by a standard curve.

### Quantitative real time-polymerase chain reaction

Total RNA was isolated from neutrophils using Trizol reagent according to the manufacturer's instructions (Invitrogen, Carlsbad, USA). The cDNA was synthesized using the Revert Aid First Strand cDNA Synthesis Kit (Roche, Basel, Switzerland). The cDNA was detected by Quantitative Real Time PCR (qRT-PCR). The specific primers (Supplementary Table 2) of selenoproteins, HSPs, inflammatory factors, GAPDH and β-actin based on known swine sequences were designed by Primer Premier Software (PREMIER Biosoft International, USA). β-actin was used as an internal reference. qRT-PCR was performed on a Light Cycler^®^480 System (Roche, Basel, Switzerland) using Fast Universal SYBR Green Master (Roche, Basel, Switzerland). Only one peak for each PCR product was shown in the melting curve analysis. The relative abundance of mRNA was calculated according to the method of 2^-ΔΔCt^ [63], accounting for gene-specific efficiencies and was normalized to the mean expression of the above-mentioned index.

### Statistical analysis

Statistical analyses of all data were performed with GraphPad Prism (version 5.0, Graph Pad Software Inc., San Diego, CA, USA). All data were analyzed by one-way ANOVA analysis, had a normal distribution and passed equal variance testing. Quantitative data are presented as the mean ± SD. Samples with different superscript letters represented statistically significant differences (*P* < 0.05).

## SUPPLEMENTARY TABLES







## Abbreviations

Nrf2: nuclear factor E2-related factor 2
ROS: reactive oxygen species
HSPs: heat shock proteins
Gpx: glutathione peroxidase
TrxR: thioredoxin reductase
Sepp1: selenoprotein P
Seln: selenoprotein N
Selw: selenoprotein W
IL-1: interlenkin-1β
IL-6: interlenkin-6
IL-10: interlenkin-10
TNF-α: tumor necrosis factor α
COX-2: cyclooxygenaase
Keap1: Kelch like-ECH-associated protein 1
ARE: antioxidant-response element
SOD2: superoxide dismutase 2
HO-1: haemoxygenase-1
ApoE: apolipoprotein E
MCP-1: monocyte chemotactic protein 1
DE: digestive energy
BW: body weight
MSHP: microspheric particles.
