# Supplementary material for: Oxidative stress induced by Se-deficient high-energy diet implicates neutrophil dysfunction via Nrf2 pathway suppression in swine
Source: Oncotarget. 2017 Jan 7;8(8):13428–39. doi: 10.18632/oncotarget.14550 (PMC5355109; doi:10.18632/oncotarget.14550)
Supplement: Supplementary file 1 [file oncotarget-08-13428-s001.pdf]

www.impactjournals.com/oncotarget/ Oncotarget Advance Publications

Oxidative stress induced by Se-deficient high-energy diet implicates neutrophil dysfunction via Nrf2 pathway suppression in swine

Supplementary Materials

Supplementary Table 1: The material and nutrient component in basal diet and high fat diet. See Supplementary_Table_1

Supplementary Table 2: Gene-special primers used in the real-time quantitative reverse-transcription PCR. See Supplementary_Table_2
