# Supplementary material for: Oxidative stress induced by Se-deficient high-energy diet implicates neutrophil dysfunction via Nrf2 pathway suppression in swine
Source: Oncotarget. 2017 Jan 7;8(8):13428–39. doi: 10.18632/oncotarget.14550 (PMC5355109; doi:10.18632/oncotarget.14550)
Supplement: Supplementary file 2 [file oncotarget-08-13428-s002.docx]

**Supplemental table 1** The material and nutrient component in basal diet and high fat diet

|  | Basal diet（g/kg） | | | High energy diet（g/kg） | | |
| --- | --- | --- | --- | --- | --- | --- |
| material | 10-30kg | 30-60kg | 60-110kg | 10-30kg | 30-60kg | 60-110kg |
| corn | 721.59 | 753.64 | 802.36 | 670.88 | 655.14 | 654.9 |
| bean pulp | 220 | 195 | 150 | 231 | 214 | 178 |
| wheat bran | 20 | 20 | 20 | 20 | 20 | 20 |
| lard |  |  |  | 30 | 70 | 110 |
| fish oil |  |  |  | 10 | 10 | 10 |
| lysine | 7.24 | 4.11 | 3.9 | 6.95 | 3.62 | 3.15 |
| methionine | 0.86 | 0.15 | 0.04 | 0.89 | 0.24 | 0.12 |
| threonine | 0.86 |  |  | 0.85 |  |  |
| tryptophan | 0.15 |  | 0.1 | 0.13 |  | 0.03 |
| salt | 3 | 3 | 3 | 3 | 3 | 3 |
| calcium carbonate | 9.8 | 10.6 | 9.6 | 9.8 | 10 | 9.3 |
| calcium hydrogen phosphate | 10.5 | 7.5 | 5 | 10.5 | 8 | 5.5 |
| gunk | 6 | 6 | 6 | 6 | 6 | 6 |
| nutrient component（%） |  |  |  |  |  |  |
| digestive energy（DE） | 3296 | 3294 | 3303 | 3492 | 3688 | 3929 |
| Fat energy / digestion |  |  |  | 16.14% | 24.05% | 30.81% |
| crude protein | 16.1 | 15 | 13.4 | 16.2 | 15 | 13.4 |
| crude fat | 3 | 3.1 | 3.2 | 6.8 | 10.7 | 14.6 |
| corase fiber | 3.1 | 3 | 2.8 | 3 | 2.9 | 2.7 |
| calcium | 0.7 | 0.65 | 0.55 | 0.7 | 0.65 | 0.55 |
| Available phosphorus | 0.3 | 0.25 | 0.21 | 0.3 | 0.25 | 0.21 |
| lysine | 1.15 | 0.92 | 0.8 | 1.15 | 0.92 | 0.8 |
| methionine | 0.34 | 0.26 | 0.23 | 0.34 | 0.26 | 0.23 |
| Methionine cysteine | 0.63 | 0.54 | 0.49 | 0.63 | 0.54 | 0.48 |
| threonine | 0.68 | 0.56 | 0.5 | 0.68 | 0.56 | 0.5 |
| tryptophan | 0.19 | 0.16 | 0.15 | 0.19 | 0.17 | 0.15 |
