# Supplementary material for: Oxidative stress induced by Se-deficient high-energy diet implicates neutrophil dysfunction via Nrf2 pathway suppression in swine
Source: Oncotarget. 2017 Jan 7;8(8):13428–39. doi: 10.18632/oncotarget.14550 (PMC5355109; doi:10.18632/oncotarget.14550)
Supplement: Supplementary file 3 [file oncotarget-08-13428-s003.docx]

**Supplemental Table 2** Gene-special primers used in the real-time quantitative reverse-transcription PCR.

| Gene | Primer sequence (5′→3′) |
| --- | --- |
| Gpx1 | Forward:GATGCCACTGCCCTCATGA |
|  | Reverse:TCGAAGTTCCATGCGATGTC |
| Gpx2 | Forward:AGAATGTGGCCTCGCTCTGA |
|  | Reverse:GGCATTGCAGCTCGTTGAG |
| Gpx3 | Forward:TGCACTGCAGGAAGAGTTTGAA |
|  | Reverse:CCGGTTCCTGTTTTCCAAATT |
| Gpx4 | Forward:TGAGGCAAGACGGAGGTAAACT |
|  | Reverse:TCCGTAAACCACACTCAGCATATC |
| TrxR1 | Forward:GATTTAACAAGCGGGTCATGGT |
|  | Reverse:CAACCTACATTCACACACGTTCCT |
| Sephs | Forward:TGGCTTGATGCACACGTTTAA |
|  | Reverse:TGCGAGTGTCCCAGAATGC |
| Sep | Forward:ACAGCCCTGCCAAGCAGAT |
|  | Reverse:AACAGGGAGGCTGGGTAACAC |
| Selh | Forward:TGGTGGAGGAGCTGAAGAAGTAC |
|  | Reverse:CGTCATAAATGCTCCAACATCAC |
| Seli | Forward:GATGGTGTGGATGGAAAGCAA |
|  | Reverse:GCCATGGTCAAAGAGTTCTCCTA |
| Selm | Forward:CAGCTGAATCGCCTCAAAGAG |
|  | Reverse:GAGATGTTTCATGACCAGGTTGTG |
| Sepp | Forward:AACCAGAAGCGCCAGACACT |
|  | Reverse:TGCTGGCATATCTCAGTTCTCAGA |
| Selt | Forward:GGCTTAATAATCGTTGGCAAAGA |
|  | Reverse:TGGCCCCATTGCCAGATA |
| Selx | Forward:ATCCCTAAAGGCCAAGAATCATC |
|  | Reverse:GGCCACCAAGCAGTGTTCA |
| SelK | Forward:CAGGAAACCCCCCTAGAAGAA |
|  | Reverse:CTCATCCACCGGCCATTG |
| Sepn | Forward:ACCTGGTCCCTGGTGAAAGAG |
|  | Reverse:AGGCCAGCCAGCTTCTTGT |
| Sepw | Forward:CACCCCTATCTCCCTGCAT |
|  | Reverse:GAGCAGGATCACCCCAAACA |
| Hsp40 | Forward: AGACCTCCAACAACATTCCAG |
|  | Reverse: TAATCCTGGCTGGGTAAATGA |
| Hsp60 | Forward: CTCATCTCACTCGGGCTTATG |
|  | Reverse: TATCACCGTCCTTCCCTTTG |
| Hsp70 | Forward: CAAAGCAGACCCAGACTTTCA |
|  | Reverse: AGAAGGTTGTTGTCCCTGGTC |
| Hsp90 | Forward: AAATCCAGACCATTCCATCATC |
|  | Reverse:TGAAGCCAGAAGACAGCAGAG |
| IL-1α | Forward:ACCCGACTGTTTGTGAGTGC |
|  | Reverse:TTCCCAGAAGAAGAGGAGACTG |
| IL-1β | Forward: TCTCCAGCCAGTCTTCATTGT |
|  | Reverse:GCCATCAGCCTCAAATAACAG |
| IL-2 | Forward: ATTGCACTAACCCTTGCACTC |
|  | Reverse:CAACTGTAAATCCAGCAGCAA |
| IL-4 | Forward: CGGCACATCTACAGACACCAC |
|  | Reverse:CTTCATGCACAGAACAGGTCA |
| IL-6 | Forward: GCTATGAACTCCCTCTCCACA |
|  | Reverse:ACCTTTGGCATCTTCTTCCAG |
| IL-8 | Forward: TGAGAAGCAACAACAACAGCA |
|  | Reverse:AGCACAGGAATGAGGCATAGA |
| IFN-γ | Forward: AGCTTTGCGTGACTTTGTGTT |
|  | Reverse:GGTCCACCATTAGGTACATCTG |
| TGF-β1 | Forward: TGTCCACCATTCATTTGTTCC |
|  | Reverse:AGGCATTCAGGATAAGGTCCA |
| Nf-κb p50 | Forward: CCATGCTGGAACCACTAAATC |
|  | Reverse: TACGGCCTCTCTGTCATCACT |
| Nf-κb p65 | Forward:GTGTGTAAAGAAGCGGGACCT |
|  | Reverse:CACTGTCACCTGGAAGCAGA |
| TNF-α | Forward:ACCAGCCAGGAGAGAGACAAG |
|  | Reverse:AGCGTGTGAGAGGGAGAGAGT |
| iNOS | Forward: ACCACGGAACCTAATGATGG |
|  | Reverse:GAGTTGGAGAGGGAGGGAGAT |
| COX-2 | Forward: TCCAAATACAACCCTGTCCTG |
|  | Reverse: CCACATCTTACCGCCTGATTA |
